# Effects of High-Mobility Group Box-1 on Mucosal Immunity and Epithelial Differentiation in Colitic Carcinoma

**DOI:** 10.3390/ijms25136846

**Published:** 2024-06-21

**Authors:** Takamitsu Sasaki, Rina Fujiwara-Tani, Yi Luo, Ruiko Ogata, Rika Sasaki, Ayaka Ikemoto, Yukiko Nishiguchi, Chie Nakashima, Shingo Kishi, Kiyomu Fujii, Hitoshi Ohmori, Naohide Oue, Hiroki Kuniyasu

**Affiliations:** 1 Department of Molecular Pathology, Nara Medical University, 840 Shijo-Cho, Kashihara 634-8521, Nara, Japan; takamitu@fc4.so-net.ne.jp (T.S.); lynantong@hotmail.com (Y.L.); pkuma.og824@gmail.com (R.O.); rika0st1113v726296v@icloud.com (R.S.); a.ikemoto.0916@gmail.com (A.I.); yukko10219102@yahoo.co.jp (Y.N.); c-nakashima@naramed-u.ac.jp (C.N.); toto1999-dreamtheater2006-sms@nifty.com (K.F.); brahmus73@hotmail.com (H.O.); 2 Pathology Laboratory, Research Institute, Tokushukai Nozaki Hospital, 2-10-50 Tanigawa, Daito 574-0074, Osaka, Japan; nmu6429@yahoo.co.jp; 3 Pathology Laboratory, Miyoshi Central Hospital, 10531 Higashi-Sakaya, Miyoshi 728-8502, Hiroshima, Japan; naoue@hiroshima-u.ac.jp

**Keywords:** ulcerative colitis, colitic carcinoma, sporadic colorectal carcinoma, HMGB1, mucosal immunity

## Abstract

Abnormalities in mucosal immunity are involved in the onset and progression of ulcerative colitis (UC), resulting in a high incidence of colorectal cancer (CRC). While high-mobility group box-1 (HMGB1) is overexpressed during colorectal carcinogenesis, its role in UC-related carcinogenesis remains unclear. In the present study, we investigated the role of HMGB1 in UC-related carcinogenesis and sporadic CRC. Both the azoxymethane colon carcinogenesis and dextran sulfate sodium colitis carcinogenesis models demonstrated temporal increases in mucosal HMGB1 levels. Activated CD8+ cells initially increased and then decreased, whereas exhausted CD8+ cells increased. Additionally, we observed increased regulatory CD8+ cells, decreased naïve CD8+ cells, and decreased mucosal epithelial differentiation. In the in vitro study, HMGB1 induced energy reprogramming from oxidative phosphorylation to glycolysis in CD8+ cells and intestinal epithelial cells. Furthermore, in UC dysplasia, UC-related CRC, and hyperplastic mucosa surrounding human sporadic CRC, we found increased mucosal HMGB1, decreased activated CD8+ cells, and suppressed mucosal epithelial differentiation. However, we observed increased activated CD8+ cells in active UC mucosa. These findings indicate that HMGB1 plays an important role in modulating mucosal immunity and epithelial dedifferentiation in both UC-related carcinogenesis and sporadic CRC.

## 1. Introduction

Patients with long-standing inflammatory bowel disease (IBD), ulcerative colitis (UC), and Crohn’s disease (CD) have increased risks of colorectal cancer (CRC). The incidence of CRC is up to 18% in a 30-year period of IBD [1]. Furthermore, CRC accounts for 15% of deaths in patients with IBD [2]. The major carcinogenic risks of UC and CD are similar, including disease duration and expansion [1]. Despite differences in molecular abnormalities between UC-associated dysplasia and sporadic CRC development, IBD-associated cancers have genetic abnormalities and microsatellite instability similar to sporadic CRC [2]. Furthermore, it is important to note that chronic persistent inflammation is a common cause of carcinogenesis in IBD-associated CRC and sporadic CRC [3]. Moreover, the prognosis of UC-related CRC is similar to that of sporadic CRC [4].

Although the details of IBD pathogenesis remain unclear, abnormalities in mucosal immunity are involved. In particular, an increase in cytotoxic CD8+ T cell number plays an important role [5,6]. UC-associated CD8+ effector T cells cause tissue destruction and produce tumor necrosis factor-α (TNFα); however, post-effector cells have lost their activity [7].

High-mobility group box-1 (HMGB1) is overexpressed in many solid cancers, including CRC, and promotes cancer proliferation, invasion, and metastasis [8,9]. Furthermore, it suppresses monocytes by inhibiting innate mucosal immunity [9]. Mucosal levels of HMGB1 levels increase during colorectal carcinogenesis [10,11]. The effects of HMGB1 on acquired immunity have also recently attracted research attention. HMGB1 is involved in T cell exhaustion and dysfunction [12]. Additionally, HMGB1 knockdown does not affect tumor cell proliferation but improves effector CD8+ T cell responses, causing tumor rejection [13]. Furthermore, HMGB1 inhibition ameliorates colitis in animal models [14]. Thus, HMGB1 plays an important role in UC and UC-related carcinogenesis.

Disorders in the intestinal flora are involved in UC onset and exacerbation [15]. Intestinal commensal bacteria, including *Fusobacterium valium*, invade the colonic epithelium and induce the secretion of inflammatory cytokines, leading to inflammatory lesions such as cryptitis and crypt abscesses in UC [16]. HMGB1 causes dysbiosis in inflammatory diseases [17]. Mucosal barriers such as mucin 2 (MUC2) and antimicrobial peptides such as α-defensin (αDEF) are secreted from the intestinal epithelium and maintain the intestinal microbiome [18,19]. However, their relationships with HMGB1 remain unclear.

Thus, while mucosal effector T cells and the intestinal flora play important roles in UC, their relationships with the transition to colitic CRC and the influence of HMGB1 on UC-associated carcinogenesis remain unclear. To investigate the relationship between HMGB1 and mucosal immunity or mucosal differentiation in UC-associated carcinogenesis, we examined mucosal immunity using four systems: a mouse colon carcinogenesis model, a mouse colitis carcinogenesis model, samples of human colitic carcinoma, and samples of human hyperplastic mucosa surrounding colon cancer.

## 2. Results

### 2.1. Effects of HMGB1 in the Azoxymethane (AOM)-Induced Mouse Colon Carcinogenesis Model

**Figure 1:**
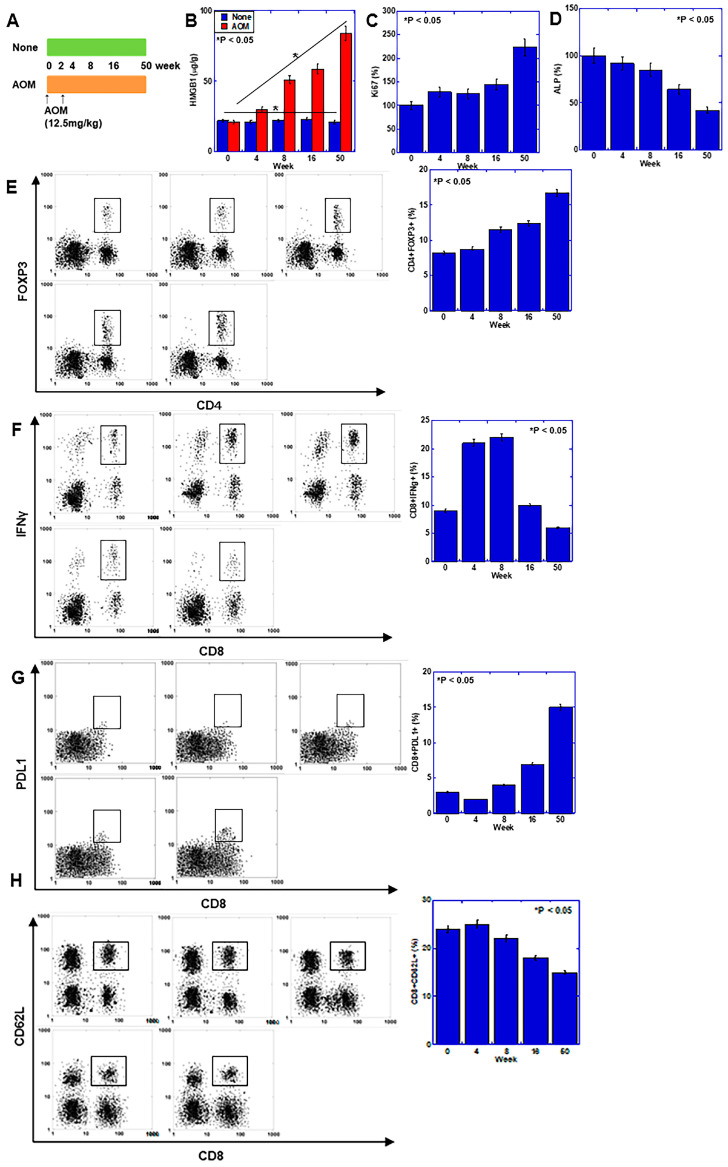
Effect of HMGB1 in the mouse AOM-induced colon carcinogenesis model. (**A**) Experimental protocol. Five BALB/c mice (male, 5 weeks of age) were injected with AOM (12.5 mg/kg) intraperitoneally (ip) twice and observed for 50 weeks. Another five mice were not injected with AOM and were observed as a parallel control. Mucosal tissues were collected for ELISA and flow cytometry. (**B**–**D**) ELISA for HMGB1 (**B**), Ki67 (**C**) and ALP (**D**). Protein levels in the AOM group were expressed as percentages of those in the non-AOM group. (**E**–**H**) Flow cytometry for Treg (CD8+FOXP3+) (**E**), activated CD8 (CD8+IFNγ+) (**F**), exhausted CD8 (CD8+PDL1+) (**G**) and naïve CD8 (CD8+CD62L+) (**H**). Error bar and standard deviation from five mice or three independent trials. Statistical differences were calculated using analysis of variance with the Bonferroni correction. HMGB1, high-mobility group box-1; AOM, azoxymethane; ALP, intestinal type alkaline phosphatase; FOXP3, forkhead box protein P3; IFNγ, interferon γ; PDL1, programmed cell death ligand-1.

After administering AOM twice to BALB/c mice, the temporal changes in the mucosa were examined and compared with those in the parallel control group (Figure 1A). The AOM group showed increased HMGB1 level and proliferative activity (K i67) over time after AOM administration compared with the parallel control (Figure 1B,C). Levels of markers of mucosal epithelial differentiation (alkaline phosphatase and alkaline phosphatase) temporally decreased (Figure 1D), while CD4+ forkhead box protein P3 (FOXP3)+ cells (regulatory T cells, Tregs) increased (Figure 1E). The levels of CD8+ Interferon (IFN)γ+ cells (activated CD8+ cells) increased at 4 and 8 weeks and then decreased (Figure 1F). In contrast, CD8+ programmed cell death ligand-1(PDL1)+ cell (exhausted CD8+ cells) levels increased after 8 weeks (Figure 1G). CD8+CD62L+ cell (naïve CD8+ cells) levels also decreased after 8 weeks (Figure 1H).

### 2.2. Effects of HMGB1 in the Mouse Dextran Sulfate Sodium (DSS) Colitis-Associated Colon Carcinogenesis Model

**Figure 2:**
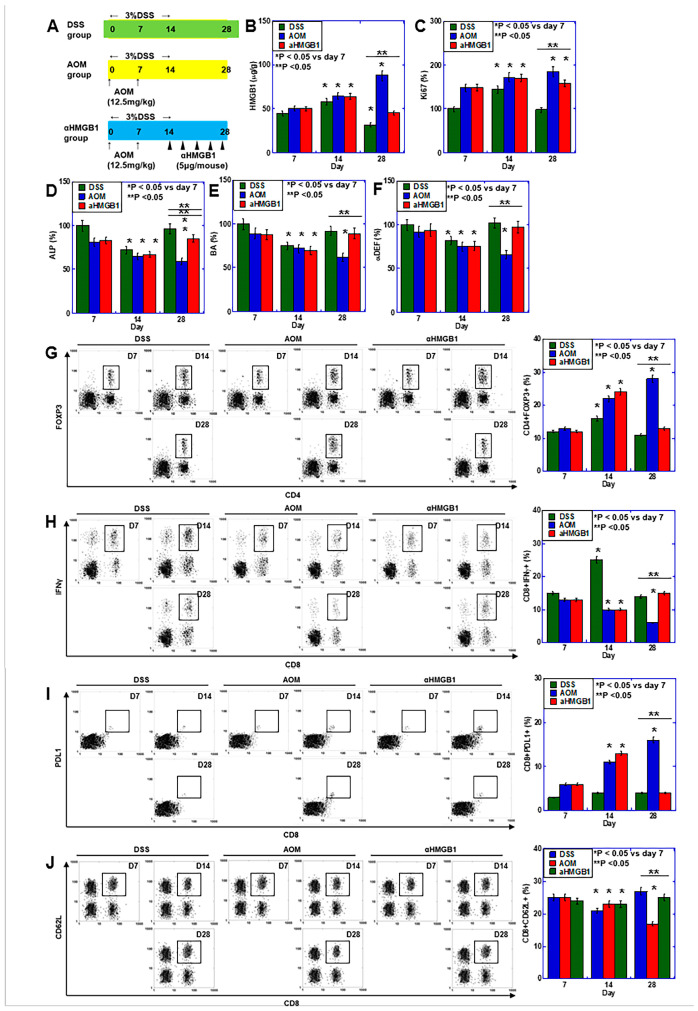
Effect of HMGB1 in the mouse dextran sulfate sodium (DSS) colitis-associated colon carcinogenesis model. (**A**) Experimental protocol. In the DSS group, five BALB/c mice (male, 5 weeks of age) were administered DSS (3%) for 2 weeks. In the AOM group, five BALB/c mice were administered DSS and injected twice intraperitoneally (ip) with AOM. In the αHMGB1 group, five BALB/c mice were administered DSS and injected twice intraperitoneally (ip) with AOM following αHMGB1 injection (5 μg/mouse) ip 5 times. All mice were observed for 4 weeks. Mucosal tissues were collected for ELISA and flow cytometry. (**B**–**F**) ELISA for HMGB1 (**B**), Ki67 (**C**), ALP (**D**), BA (**E**), and αDEF (**F**). (**G**–**J**) Flow cytometry for Treg (CD8+FOXP3+) (**G**), activated CD8 (CD8+IFNγ+) (**H**), exhausted CD8 (CD8+PDL1+) (**I**) and naïve CD8 (CD8+CD62L+) (**J**). Error bars: standard deviation from five mice and three independent trials. Statistical differences were calculated using analysis of variance with Bonferroni correction. DSS, dextran sulfate sodium; HMGB1, high-mobility group box-1; αHMGB1, anti-HMGB1 antibody; AOM, azoxymethane; ALP, intestinal type alkaline phosphatase; FOXP3, forkhead box protein P3; IFNγ, interferon γ; PDL1, programmed cell death ligand-1; BA, butyric acid; αDEF, α-defensin.

As reported above, in the AOM colorectal carcinogenesis model, the increase in the HMGB1 level was accompanied by decreased epithelial differentiation, a decrease in activated CD8+ cells and naïve CD8+ cells, and an increase in exhausted CD8+ cells. Next, we investigated the role of HMGB1 in colitis carcinogenesis using a mouse model of DSS colitis combined with AOM. In this study, HMGB1 expression was suppressed using an anti-HMGB1 antibody. We observed increased mucosal HMGB1 levels in all groups after DSS administration, which subsequently decreased in the DSS group. In contrast, the HMGB1 levels continued to increase in the AOM group. In the αHMGB1 group, HMGB1 levels decreased after the start of antibody administration (Figure 2B). Proliferative activity increased with DSS administration in all three groups but subsequently decreased in the DSS group and continued to increase in the AOM group. We observed partial inhibition in the αHMGB1 group (Figure 2C). Alkaline phosphatase (ALP) levels decreased following DSS administration, but recovered in the DSS and αHMGB1 groups, and further decreased in the AOM group (Figure 2D). The levels of the healthy intestinal flora marker (butyric acid, BA) and αDEF decreased only on day 14 in the DSS group, decreased temporally in the AOM group, and returned to normal after antibody administration in the αHMGB1 group (Figure 2E,F).

Tregs increased on day 28 in the AOM group (Figure 2G). Activated CD8+ cells increased only on day 14 in the DSS group, decreased temporally in the AOM group, and returned to normal after antibody administration in the αHMGB1 group (Figure 2H). In contrast, exhausted CD8+ cells decreased only on day 14 in the DSS group, increased temporally in the AOM group, and returned to normal after antibody administration in the αHMGB1 group (Figure 2I). Naive CD8+ cells decreased only on day 14 in the DSS group, decreased temporally in the AOM group, and returned to normal after antibody administration in the αHMGB1 group (Figure 2J).

Thus, in the DSS colitis carcinogenesis model, an increase in HMGB1 was associated with decreased epithelial differentiation, impaired intestinal flora, a decrease in activated CD8+ and naïve CD8+ cells, and an increase in exhausted CD8+ and Treg cells. These changes were abrogated by the anti-HMGB1 antibody.

### 2.3. Effects of HMGB1 on Lymphocytes Using In Vitro CD8+ Cell Activation Assay

**Figure 3:**
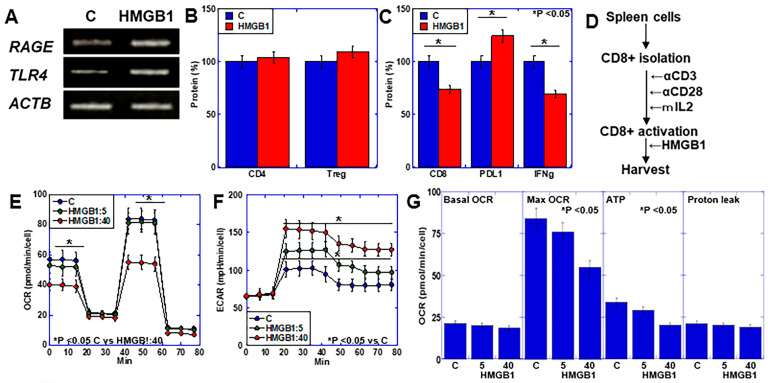

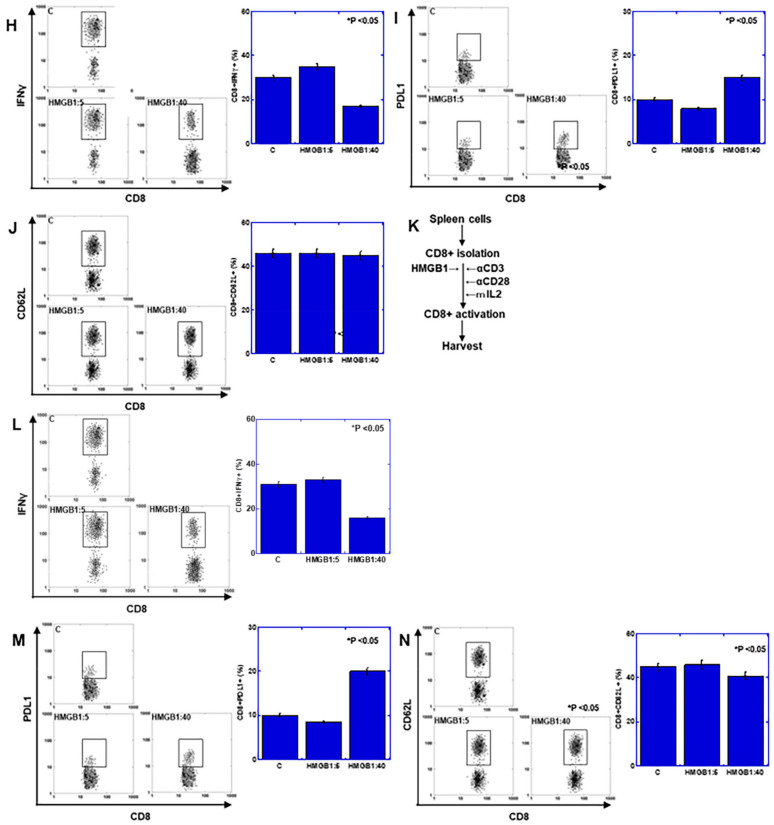
Effect of HMGB1 on lymphocytes using in vitro CD8 cell activation assay. (**A**–**C**) Lymphocytes separated from spleen cells were subjected to RT-PCR and ELISA. Lymphocytes were treated with HMGB1 (10 μg/mL, 48 h). (**A**) Gene expression levels of RAGE and TLR4. (**B**) Protein levels of CD4 and Treg. (**C**) Protein levels of CD8, PDL1, and IFNγ. (**D**–**J**) CD8+ cell activation in vitro following HMGB1 treatment (5 or 40 μg/mL, 48 h). (**D**) Experimental protocol. (**E**) Mitochondrial stress assay. (**F**) Glycolytic stress assay. (**G**) OXPHOS parameters. (**H**–**J**) Flow cytometry for activated CD8 (CD8+IFNγ+) (H), exhausted CD8 (CD8+PDL1+) (**I**) and naïve CD8 (CD8+CD62L+) (**J**). (**K**–**N**) CD8+ cell activation in vitro with concurrent HMGB1 treatment (5 or 40 μg/mL). (**K**) Experimental protocol. (**L**–**N**) Flow cytometry for activated CD8 (CD8+IFNγ+) (**L**), exhausted CD8 (CD8+PDL1+) (**M**) and naïve CD8 (CD8+CD62L+) (**N**). Error bars: standard deviation from three independent trials. Statistical differences were calculated using analysis of variance with the Bonferroni correction. HMGB1, high-mobility group box-1; RT-PCR, reverse transcription–polymerase chain reaction; ELISA, enzyme-linked immunosorbent assay; RAGE, receptor for advanced glycation end products; TLR4, Toll-like receptor 4; ACTB, β-actin; Treg, regulatory T cell; IFNγ, interferon γ; PDL1, programmed cell death ligand-1; OXPHOS, oxidative phosphorylation; OCR, oxygen consumption rate; ECAR, extracellular acidification rate.

After spleen cells were collected and lymphocytes were isolated, the following were observed: increased expression of HMGB1 receptors, receptor for advanced glycation end products (RAGE), and Toll-like receptor 4 (TLR4) following HMGB1 treatment (Figure 3A). While HMGB1 treatment did not affect CD4 or FOXP3 protein levels (Figure 3B), CD8 and IFNγ protein levels decreased, and PDL1 protein levels increased (Figure 3C).

Next, CD8+ T cells were isolated from spleen cells and activated in vitro, and then treated with HMGB1 at 5 or 40 μg/mL (Figure 3D). We observed HMGB1 concentration-dependent suppression of oxidative phosphorylation (OXPHOS) and the enhancement of glycolysis (Figure 3E,F), as well as decreased mitochondrial respiration and ATP production (Figure 3G). At 5 μg/mL of HMGB1, activated CD8+ cells (IFNγ+) increased while those of exhausted CD8+ cells (PDL1+) decreased (Figure 3H). In contrast, at 40 μg/mL of HMGB1, activated CD8+ cells decreased while exhausted CD8+ cells increased (Figure 3I). Naïve CD8+ cells (CD62L+) remained unchanged, regardless of the HMGB1 concentration (Figure 3J).

In contrast, HMGB1 treatment during CD8 activation demonstrated changes in activated CD8+ cells and exhausted CD8+ cells similar to those in post-activation HMGB1 treatment, while naïve CD8+ cells decreased at both HMGB1 concentrations. Thus, HMGB1 may promote CD8 activation at low concentrations but promotes exhaustion at high concentrations. Furthermore, HMGB1 may inhibit naïve CD8+ cells regardless of the HMGB1 concentration.

### 2.4. Effects of HMGB1 on Intestinal Epithelial Cells

**Figure 4:**
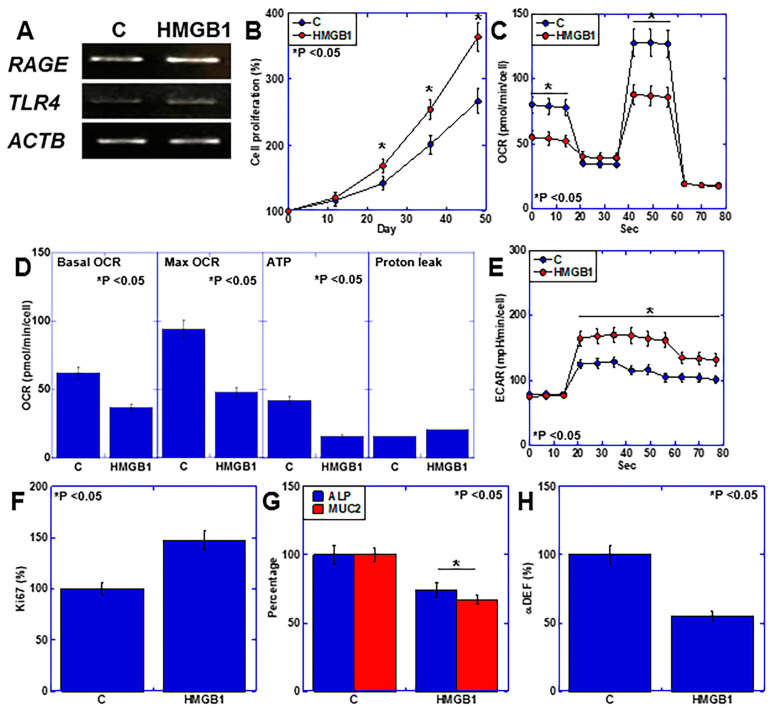
Effects of HMGB1 on intestinal epithelial cells. Mouse IEC6 intestinal epithelial cells were treated with HMGB1 (20 μg/mL, 48 h) to assess the effects of HMGB1. (**A**) RAGE and TLR4 gene expression levels. (**B**) Cell growth analysis. (**C**) Mitochondrial stress assays. (**D**) OXPHOS parameters. (**E**) Glycolytic stress assay. (**F**) Ki67 (proliferation). (**G**) MUC2 and ALP expression (intestinal epithelial differentiation). (**H**) DEF (intestinal flora maintenance). Error bars: standard deviation from three independent trials. Statistical differences were calculated using analysis of variance with the Bonferroni correction. HMGB1, high-mobility group box-1; RAGE, receptor for advanced glycation end products; TLR4, Toll-like receptor 4; ACTB, β-actin; OXPHOS; oxidative phosphorylation; OCR, oxygen consumption rate; ECAR, extracellular acidification rate; MUC2, mucin 2; ALP, alkaline phosphatase; αDEF, α-defensin.

Next, we investigated the direct effects of HMGB1 on the intestinal epithelium of IEC6 mouse intestinal epithelial cells (Figure 4). Both RAGE and TLR4 were expressed in IEC6 cells, and their expression was promoted by HMGB1 treatment (Figure 4A). HMGB1 also promoted IEC6 cell proliferation (Figure 4B). Examination of the energy metabolism revealed that HMGB1 suppressed OXPHOS (Figure 4C), mitochondrial respiration and ATP production (Figure 4D) but promoted glycolysis (Figure 4E). Additionally, HMGB1 increased Ki67 but decreased intestinal epithelial differentiation (MUC2 and ALP) and suppressed antimicrobial peptide production (αDEF) (Figure 4F–H).

### 2.5. Role of HMGB1 in Human UC-Associated Carcinogenesis

**Figure 5:**
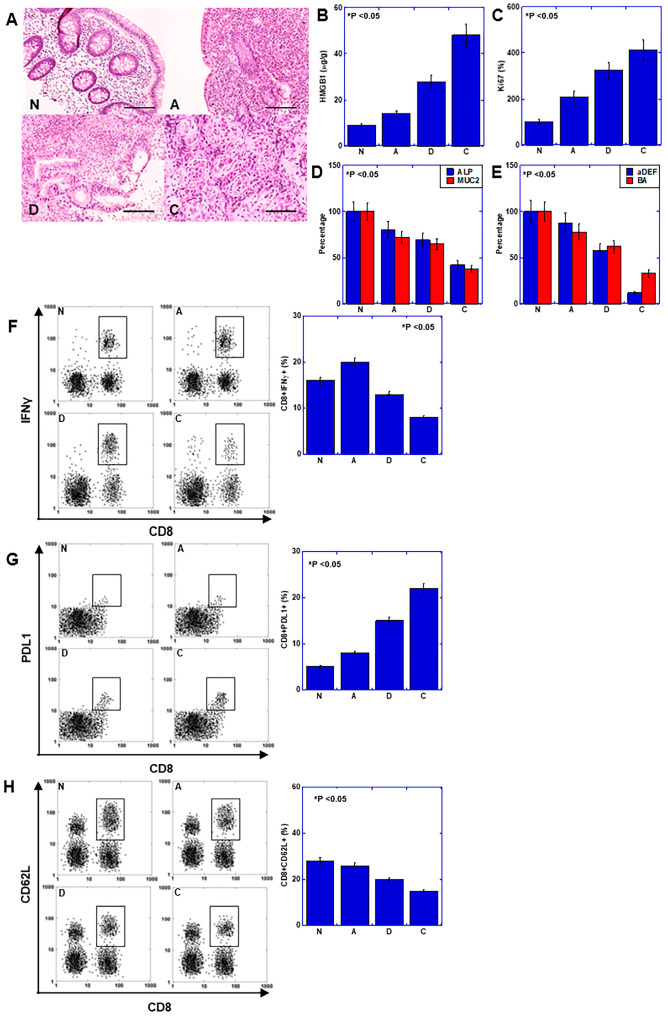
Role of HMGB1 in human UC-associated carcinogenesis. Investigation of the role of HMGB1 in mucosal immunity and epithelial differentiation in biopsy specimens from five cases of UC-associated CRC. (**A**) Histological appearance of inactive mucosa (N), active mucosa (**A**), dysplasia (**D**), and cancer (**C**). Scale bar = 100 μm. (**B**–**E**) Protein levels assessed by ELISA. (**B**) HMGB1. (**C**) Ki67 expression (proliferation). (**D**) ALP and MUC2 levels (epithelial differentiation). (**E**) DEF and BA (intestinal flora maintenance). (**F**–**H**) Flow cytometry for activated CD8 (CD8+IFNγ+) (**F**), exhausted CD8 (CD8+PDL1+) (**G**) and naïve CD8 (CD8+CD62L+) (**H**). Error bars = standard deviation of five cases and three independent trials. Statistical differences were calculated using analysis of variance with Bonferroni correction. UC, ulcerative colitis; HMGB1, high-mobility group box-1; N, non-active mucosa; A, active UC mucosa; D, UC-related dysplasia; C, UC-related carcinoma; MUC2, mucin 2; ALP, alkaline phosphatase; αDEF, α-defensin; BA, butyric acid; IFNγ, interferon γ; PDL1, programmed cell death ligand-1.

Next, we investigated the effects of HMGB1 on colonic mucosal immunity and the mucosal epithelium, which were observed in the mouse models and culture systems described above in five human cases with UC-related CRC (Figure 5). All cases included metachronous biopsy specimens with disease progression (Table 1 and Figure 5A) showing inactive mucosa (N), active mucosa (A), dysplasia (D), and cancer (C). With disease progression, HMGB1 and Ki67 levels increased (Figure 5B,C), the degree of mucosal epithelial differentiation (MUC2 and ALP) decreased (Figure 5D), and levels of antimicrobial peptide (αDEF) and healthy intestinal flora marker (BA) decreased (Figure 5E). The numbers of activated CD8+ cells (CD8+IFNγ+) increased in A but decreased in D and C (Figure 5F). In contrast, the numbers of exhausted CD8+ cells (CD8+PDL1+) were increased from A to C (Figure 5G). Conversely, the number of naïve CD8+ cells (CD8+CD62L+) decreased with disease progression (Figure 5H). These results suggest that the effects of HMGB1 on suppressing mucosal immunity and the dedifferentiation of the mucosal epithelium can be extrapolated to human UC.

### 2.6. Effects of HMGB1 on Hyperplastic Mucosa Surrounding CRC

**Figure 6:**
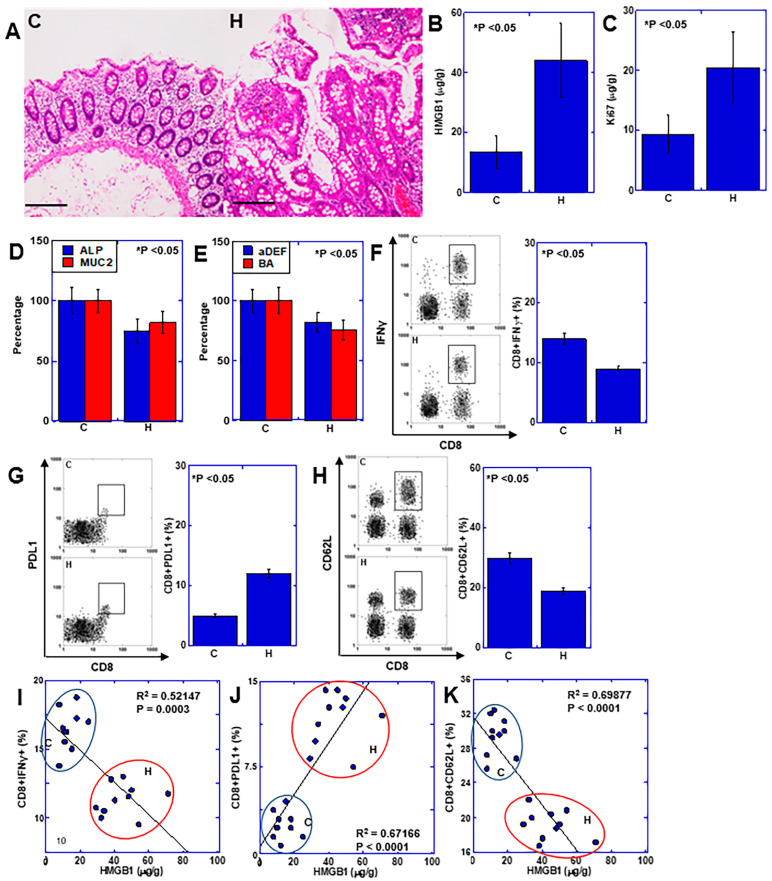
Effects of HMGB1 on hyperplastic mucosa surrounding CRC. Investigation of the role of HMGB1 in mucosal immunity and epithelial differentiation using biopsy specimens from 10 cases of CRC with hyperplastic mucosa surrounding the cancer lesions. (**A**) Histological appearance of the mucosa distant from CRC focus (C) and the hyperplastic mucosa surrounding CRC focus (H). Scale bar = 100 μm. (**B**–**E**) Protein levels of HMGB1 (**B**), Ki67 (proliferation) (**C**), ALP and MUC2 levels (epithelial differentiation) (**D**), and DEF and BA (intestinal flora maintenance) (**E**). (**F**–**H**) Flow cytometry for activated CD8 (CD8+IFNγ+) (**F**), exhausted CD8 (CD8+PDL1+) (**G**) and naïve CD8 (CD8+CD62L+) (**H**). (**I**–**K**) Relationships between HMGB1 and activated CD8 cells (CD8+IFNγ+) (**I**), or exhausted CD8 (CD8+PDL1+) (**J**), or naïve CD8 (CD8+CD62L+) (**K**). (**I**–**K**) Dots represent each case of cancer (C) and hyperplastic mucosa (H). Error bars: standard deviation of 10 cases and three independent trials. Statistical differences were calculated using analysis of variance with Bonferroni correction. Regression analysis was performed using Spearman’s correlation test. HMGB1, high-mobility group box-1; C, control non-hyperplastic mucosa distant from cancer lesion (more than 5 cm); H, hyperplastic mucosa surrounding cancer lesion (within 1 cm); MUC2, mucin 2; ALP, alkaline phosphatase; αDEF, α-defensin; BA, butyric acid; IFNγ, interferon γ; PDL1, programmed cell death ligand-1.

Our findings suggested that HMGB1 suppresses mucosal immunity in UC-related colon carcinogenesis, causes intestinal flora dysbiosis, and promotes carcinogenesis. We also investigated whether these effects of HMGB1 also occur in sporadic CRC (Figure 6). Hyperplastic mucosa surrounds CRC lesions and correlates with the malignant phenotype of CRC [22,23]. This study was performed using 10 patients with sporadic CRC with hyperplastic mucosa (Table 2 and Figure 6A). We observed HMGB1 overexpression and increased proliferative metaplasia (Ki67) in the hyperplastic mucosa (H) compared with mucosa distant from the colon cancer (C) (Figure 6B,C). The degree of mucosal epithelial differentiation (MUC, ALP) and levels of antibacterial peptide (αDEF), and healthy intestinal flora marker (BA) were decreased (Figure 6D,E). In mucosal immunity, the hyperplastic mucosa showed decreased numbers of activated CD8 cells (CD8+IFNγ+), and increased numbers of exhausted T cells (CD8+PDL1+) (Figure 6F,G). In addition, the hyperplastic mucosa showed decreased numbers of naïve T cells (CD8+CD62L+) (Figure 6H). Examination of the relationship between HMGB1 and activated, exhausted and naïve CD8+ cells, revealed that activated and naïve CD8+ cells decreased inversely and were correlated with HMGB1, while exhausted CD8+ cells increased in correlation with HMGB1 (Figure 6I–K). Thus, HMGB1 also suppressed mucosal immunity and inhibited mucosal epithelial differentiation in sporadic CRC, similar to its effects in UC.

## 3. Discussion

In this study, we analyzed the effects of HMGB1 on mucosal immunity in the colon using mouse AOM carcinogenesis models, mouse DSS carcinogenesis models, human UC and UC-associated CRC samples, and samples of mucosa surrounding human colon cancer. Our findings demonstrated that HMGB1 is overexpressed in the dedifferentiated mucosa of premalignant states and that the reprogramming of the energy metabolism of CD8+ lymphocytes in the mucosa leads to decreased naïve CD8 counts and effector CD8 cell exhaustion, thereby suppressing antitumor mucosal immunity.

Analysis of UC cases showed increased CD8+IFNγ+ cells during the inflammatory process. In contrast, during the carcinogenic process, CD8+IFNγ+ cells and CD8+CD62L+ cells decreased while CD8+PDL1+ cells (exhausted CD8+ cells), increased. Thus, in mouse models, HMGB1 may lead to effector T cell exhaustion during carcinogenesis, further suppressing naïve CD8+ cells. A previous study in a mouse model of colitis carcinogenesis reported increased numbers of CD8+ IFNγ+ T cells during the inflammatory process, with decreased levels in the tumor and surrounding mucosa [24]. Thus, changes in effector T cells occur during the transition from an inflammatory to a carcinogenic process. Our data showed that lymphocyte alterations in colitis carcinogenesis paralleled HMGB1 levels, which may play an important role in this process. In our in vitro study, HMGB1 reprogrammed the energy metabolism of both activated CD8+ and mucosal epithelial cells, thus altering their properties.

Energy metabolism in lymphocytes differs depending on their differentiation status [25]. While naïve T cells produce energy through OXPHOS, effector T cells rely on glycolysis [26]. Furthermore, progenitor-exhausted T cells depend on OXPHOS, whereas terminally exhausted T cells depend on glycolysis [27]. Our findings suggest that HMGB1 activates effector T cells by suppressing OXPHOS and promoting glycolysis; however, long-term stimulation may ultimately induce exhaustion and reduce mucosal antitumor immunity. Furthermore, the findings suggest that CD62L expression decreased with increasing HMGB1 levels. Naive CD8+ T cells rely on fatty acid oxidation as their primary energy source [28]. Thus, the HMGB1 suppression of OXPHOS may lead to the suppression of naïve T cells.

In the present study, we observed increased Treg counts with increasing mucosal HMGB1 levels in both mouse models and human cases. In patients with coronavirus pneumonia, an increase in T cell immunoreceptor with immunoglobulin and ITIM domain (TIGIT)+ Treg cells is correlated with plasma HMGB1 levels [29]. HMGB1 also increases Treg counts in CRC [30]. However, in patients with idiopathic thrombocytopenia, HMGB1 expression leads to a decrease in the Treg number [31,32]. Our data also showed that HMGB1 treatment of splenocytes reduced the number of Tregs. This suggests that the increase in the number of mucosal Tregs observed in our study was not a direct effect of HMGB1. In contrast, the energy metabolism of Tregs mainly involves OXPHOS through the uptake of extracellular lactate [33,34]. Thus, HMGB1 may promote glycolytic energy metabolism in epithelial and CD8+ T cells, and increased lactate in the microenvironment may activate Tregs. Furthermore, the dysbiosis of the intestinal flora is associated with increased Treg count [35]. This suggests that the induction of dysbiosis by HMGB1 indirectly increases Treg counts.

In the active inflammatory stage of UC, we observed mucosal dedifferentiation, as well as HMGB1 increased expression and suppressed antitumor mucosal immunity. We observed similar changes in the hyperplastic mucosa surrounding the tumor. In patients with UC, these alterations are associated with increased dysplasia severity and cancer. These results suggest that HMGB1 promotes both colitis and sporadic colon carcinogenesis by suppressing mucosal antitumor immunity and inducing epithelial dedifferentiation. Neutralizing HMGB1 expression with the specific antibody reduced mucosal epithelial dedifferentiation in the DSS carcinogenesis model, suggesting that HMGB1 suppressed mucosal epithelial differentiation. Previous studies have reported the suppressive effects of HMGB1 on epithelial differentiation, including the suppression of organoid formation in the alveolar epithelium [36], and that HMGB1-associated stemness retention in the mucosal epithelia prevents differentiation [37]. In our study, HMGB1 also caused the reprogramming of energy metabolism from OXPHOS to glycolysis in intestinal epithelial cells. In epithelial systems, differentiation and OXPHOS are linked, and the transition from OXPHOS to glycolysis promotes dedifferentiation [38,39].

In our study, HMGB1 suppressed OXPHOS and promoted glycolysis in the mucosal epithelium, thereby inhibiting differentiation, decreasing antimicrobial peptide levels, and inducing dysbiosis. MUC2 forms the mucus barrier of the intestinal mucosa, and antimicrobial peptides are involved in suppressing the bacterial invasion of the mucosal epithelium and maintaining the intestinal flora [18,19]. We observed decreased BA levels owing to dysbiosis. As BA suppresses HMGB1 expression [40], BA reduction promotes the effects of HMGB1. Dysbiosis affects UC onset and exacerbation [15]; one explanation for this effect may be an increase in the endotoxins caused by dysbiosis [41]. The intestinal flora sends signals to the mitochondria of mucosal epithelial and immune cells, resulting in altered mitochondrial metabolism, immune cell activation, induced inflammasome signaling, and altered epithelial barrier function [41]. Furthermore, the dysbiosis associated with chronic inflammation damages the mitochondria of mucosal cells, suppresses mucosal immunity, and is associated with the promotion of carcinogenesis [41]. HMGB1 influences the relationship between the intestinal microbiome, mucosal epithelium, and mucosal immunity in both UC and sporadic colon cancer, and may promote the exacerbation of UC and carcinogenesis.

We examined the differences between non-colitis and colitis carcinogenesis using two pairs of systems: a mouse non-colitis carcinogenesis model and a mouse colitis carcinogenesis model to evaluate sporadic and UC-related CRC. We observed a modest increase in HMGB1 and increased CD8+IFNγ+ cells during the colitis inflammatory process (DSS and UC) and in the early period after AOM administration, whereas CD8+IFNγ+ cells decreased and CD8+PDL1+ cells increased during carcinogenesis progression. We observed similar immunosuppression in the mucosa surrounding the cancer cells and in the late period after AOM administration. Thus, although the timing of non-colitis and colitis carcinogenesis differs, almost parallel changes are observed, and HMGB1 plays an important role in both processes. Differences in mucosal HMGB1 concentrations and duration may be related to changes in mucosal immunity from promotion to suppression. The results of our in vitro experiments revealed that low HMGB1 levels enhanced CD8+IFNγ+ cells, whereas high HMGB1 levels decreased CD8+IFNγ+ cells and increased CD8+PDL1+ cells. Based on these findings, HMGB1 is an indicator of the immune status of UC [42] and a marker of UC-related carcinogenesis [43]. Additionally, UC can be improved by suppressing HMGB1 expression [44,45]. Targeting HMGB1 is expected to contribute to the normalization of mucosal immunity, not only in UC and UC-related carcinogenesis but also in sporadic CRC.

What is lacking in our study is the identification of changes in CD4+ lymphocytes and innate immunity, and changes in the bacterial species of the intestinal flora. In particular, the fact that HMGB1 has an inhibitory effect on the monocyte system suggests that HMGB1 may be involved in such changes. This is a topic for future investigation. We used young male mice in the mouse colitic carcinogenesis model. It is interesting to see the effects of sex differences and age on mucosal immunity in animal models. Sex differences have also been reported in CD [1,2], and it is possible that the hormonal environment may have some effect. In addition, IBD generally develops at a young age, so it would be interesting to see the effects of age differences with sporadic CRCs, which usually develop in old age. In this study, we were unable to examine sex and age differences, but future animal experiments and studies using clinical specimens under the conditional sampling are necessary.

Overall, our findings demonstrate the important role that HMGB1 plays in modulating mucosal immunity and epithelial dedifferentiation in both UC-related carcinogenesis and sporadic CRC. These findings suggest that HMGB1 is secreted during carcinogenesis or from cancer cells and plays an important role in changing mucosal immunity or intestinal flora to a cancer-promoting environment. Targeting HMGB1 from the early stages of carcinogenesis is useful for suppressing carcinogenesis and malignant phenotypes of cancer.

## 4. Materials and Methods

### 4.1. Animals

The animal studies were conducted in accordance with the institutional guidelines approved by the Committee for Animal Experimentation of Nara Medical University, Kashihara, Japan, following the current regulations and standards of the Japanese Ministry of Health, Labor, and Welfare (approval nos. 9559, 11365, 11528, 11569, 11 December 2009). BALB/c mice (male, 5 weeks old) were purchased from SLC (Shizuoka, Japan). The animals were maintained in a pathogen-free animal facility at 23 °C and 50% humidity, with a 12 h light/dark cycle, and were fed a CE-2 standard diet (CLEA Japan, Inc., Tokyo, Japan). The animals were allowed to acclimate to their housing for seven days before the start of the experiment.

### 4.2. Mouse AOM-Induced Colon Carcinogenesis Model

This study used BALB/c mice (male, 5 weeks of age, 30 mice in each group; SLC Japan). Mice in the AOM group were injected with AOM (12.5 mg/kg) intraperitoneally (ip) on days 1 and 15. At each time point (weeks 0, 4, 8, 16, and 50 after the first AOM injection), five mice were euthanized using isoflurane anesthesia.

### 4.3. Mouse DSS-Related Colitis Model

This study used BALB/c mice (male, 5 weeks old, 30 mice in each group; SLC Japan). The mice were administered DSS (3%, free drink, for 14 days; MP Biomedicals, Irvine, CA, USA). Mice in the AOM and αHMGB1 groups were injected with AOM (12.5 mg/kg) ip on days 1 and 8. Mice in the αHMGB1 group were administered the anti-HMGB1 antibody (0.5 μg/mouse, R&D Systems, Minneapolis, MN, USA) ip five times between days 15 and 28. The mice were euthanized using isoflurane anesthesia on day 28.

### 4.4. Colon Specimen Preparation

After euthanasia, the entire colon was removed, flushed with normal saline, and opened from the cecum to the anus. For analysis of protein production, the colon mucosae were scraped, frozen in liquid nitrogen, and stored at −80 °C for further analysis.

### 4.5. Protein Extraction

To prepare whole-cell lysates, the cells were washed twice with cold phosphate-buffered saline (PBS) and harvested. Colonic mucosae were washed with cold PBS and pelleted using a sonicator (QSONICA, WakenBtech Co., Ltd., Kyoto, Japan). Cells or tissues were lysed using a radioimmunoprecipitation assay buffer (RIPA) containing 0.1% sodium dodecyl sulfate (SDS) (Thermo Fisher, Tokyo, Japan) [10]. Protein assays were performed using a Protein Assay Rapid Kit (Wako Pure Chemical Corporation, Osaka, Japan).

### 4.6. Enzyme-Linked Immunosorbent Assay

Enzyme-linked immunosorbent (ELIS)A kits were used according to the manufacturer’s instructions to measure protein levels of CD45RO, CD20, CD3, CD4, CD8, Foxp3 (Treg), interferon–gamma (IFNγ), CD62L, alkaline phosphatase (ALP), MUC2, α-defensin, and butyric acid in whole-cell lysates (Table 3).

### 4.7. Cell Line

The IEC6 mouse intestinal epithelial cell line was a gift from Professor Isaiah J Fidler (MD Anderson Cancer Center, TX, USA). Cells were cultured in Dulbecco’s modified Eagle’s medium (DMEM, Wako) supplemented with 10% fetal bovine serum (Sigma-Aldrich Chemical Co., St. Louis, MO, USA) at 37 °C in 5% CO_2_.

### 4.8. Flow Cytometry

To demonstrate subsets of CD8+ T cells among the colon mucosa of mouse models, human tissues, and selected spleen cells, cell surface markers were analyzed by flow cytometry (FACSCalibur, Becton Dickinson, Franklin Lakes, NJ, USA). Mouse colon mucosa was scraped by razor blades and washed with cold PBS. Human mucosa was separated from submucosal tissue by razor blades under a stereomicroscopy (SZ61, Olympus, Tokyo, Japan). Then, mucosal tissues were squeezed through a 70 μm strainer (Corning, NY, USA) and filtered through a 30 μm pre-separation filter (Miltenyi Biotech, Bergisch Gladbach, Germany). Single-cell suspensions of cells in phosphate-buffered saline (PBS) were exposed to antibodies directly coupled with a fluorochrome for 30 min on ice. The antibodies used were listed in Table 4.

### 4.9. Spleen Cell Isolation

The spleen was minced in 5 mL Hanks’ Balanced Salt Solution (HBSS, WAKO) using a scalpel. A DNase (20 μg/mL, Sigma) solution containing 1% FBS (WAKO) was added to the HBSS, and the cells were incubated at 37 °C for 30 min at room temperature. Ethylenediaminetetraacetic acid (EDTA) (1 mM; WAKO) was added to stop enzymatic reactions. The cell suspension was then filtered using a 0.22 μm filter (Merck, Tokyo, Japan) and the cells were washed three times with 10 mL of PBS. The suspension was then centrifuged at 500× *g* for 5 min at 4 °C and the supernatant was discarded. Red blood cells were lysed using red blood cell lysis buffer (Funakoshi, Tokyo, Japan). The remaining spleen cells were centrifuged at 500× *g* for 5 min at 4 °C and resuspended in cold PBS (1 × 10^8^/mL).

### 4.10. Reverse Transcription–Polymerase Chain Reaction (RT-PCR)

RT-PCR to assess human and murine mRNA expression was performed using 0.5 µg total RNA extracted from cells using an RNeasy kit (Qiagen, Germantown, MD, USA). The primer sets are listed in Table 5 and were synthesized by Sigma Genosys (St. Louis, MO, USA). The PCR products were electrophoresed on a 2% agarose gel and stained with ethidium bromide. *ACTB* mRNA was used as an internal control.

### 4.11. In Vitro Activation of CD8+ Cells

Spleen cells were passed through a SepMate-50 column (Stemcell Technologies, Cambridge, MA, USA) in SepMate Medium (Stemcell). After preparation, the cells were resuspended at 5 × 10^7^ cells/mL in the SepMate Medium. CD8+ T cells were isolated from the resuspended cells using the EasySep Mouse CD8+ T Cell Isolation Kit (Veritas, Santa Clara, CA, USA) according to the manufacturer’s instructions. The CD8+ T cells were stimulated in vitro with plate-bound anti-CD3 (2 μg/mL; BD Biosciences, Franklin Lakes, NJ, USA) and soluble anti-CD28 (1 μg/mL; BD Biosciences) and expanded in culture medium containing mouse IL2 (10 ng/mL, BioLegend, San Diego, CA, USA) for 4 days.

### 4.12. Patient Information

As written informed consent was not obtained from the patients for their participation in the present study, all identifying information was removed from patient samples before analysis to ensure strict privacy protection (unlinkable anonymization). All procedures were performed according to the Ethical Guidelines for Human Genome/Gene Research enacted by the Japanese Government, and with the approval of the Ethics Committee of Nara Medical University (approval number: 937, 20 October 2014).

### 4.13. Patients with Sporadic CRC

Frozen tissue samples were obtained from 10 patients with sigmoid colon cancer diagnosed at the Department of Molecular Pathology, Nara Medical University, between 2015 and 2022 (Table 2). After confirming hyperplastic changes in the mucosa surrounding the cancer lesion according to our previous report [20], 5 mm of mucosa was collected from the mucosa surrounding the cancer lesion (within 1 cm of the cancer) and from the non-hyperplastic mucosa distant from the cancer lesion (>5 cm). The tissues were frozen in liquid nitrogen and stored at −80 °C for further analysis.

### 4.14. Patients with UC

We selected patients diagnosed with UC at the Department of Molecular Pathology, Nara Medical University between 2015 and 2022 (Table 1). Among these, five cases were selected, which had complete sets of biopsy specimens and frozen tissues from different stages of disease progression, including inactive mucosa, active mucosa, dysplasia, and colorectal cancer. Normal mucosa was confirmed by HE staining. The fresh tissues were frozen in liquid nitrogen and stored at −80 °C for further analysis.

### 4.15. Statistical Analysis

Statistical significance was calculated using ordinary analysis of variance (ANOVA) with Bonferroni correction and Spearman’s correlation test using InStat software version 3.1 (GraphPad, Los Angeles, CA, USA). Statistical significance was set at *p* < 0.05.

## Figures and Tables

**Table 1:** Patients of ulcerative colitis.

Case#	Sex	Age	Disease	Subtype	Cancer					
			Duration ^(1)^		Site ^(2)^	Histology ^(3)^	pT ^(2)^	pN ^(2)^	M ^(2)^	pStage ^(2)^
1	F	38	16	Pancolitis	S	tub1	pT3	pN0	M0	IIA
2	M	44	24	Pancolitis	R	tub1	pT3	pN0	M0	IIA
3	F	41	20	Left colon-rectal	S	tub2	pT3	pN1	M0	IIB
4	F	50	28	Pancolitis	S	tub2	pT2	pN0	M0	I
5	M	39	18	Pancolitis	R	tub2	pT3	pN0	M0	IIA

^(1)^ Time from the diagnosis of ulcerative colitis to the detection of colon cancer (years). ^(2)^ Clinicopathological classification is according to UICC-TNM Classification [20]. transverse colon; S, sigmoid colon; R, rectum; pT2, tumor indading to muscularis propria layer; pT3, tumor invading to subserosal layer; pN0, no nodal metastasis; pN1, within three metastasized nodes; M0, no distant metastasis. ^(3)^ Histopathological differentiations are according to the Japanese Classification of Colorectal Carcinoma [21]; tub1, well-differentiated adenocarcinoma; tub2, moderately differentiated adenocarcinoma.

**Table 2:** Sporadic CRC patients.

Case#	Sex	Age	Site ^(1)^	Histology ^(2)^	pT ^(1)^	pN ^(1)^	M ^(1)^	pStage ^(1)^
1	F	94	A	por	pT3	pN1	M0	IIIB
2	M	66	S	tub2	pT4b	pN2	M0	IIIC
3	M	71	S	tub2	pT4b	pN1	M0	IIIC
4	F	83	A	tub1	pT4a	pN1	M0	IIIB
5	F	61	C	tub2	pT4a	pN1	M0	IIIB
6	M	67	R	muc	pT4a	pN1	M0	IIIB
7	F	62	S	tub2	pT3	pN1	M0	IIIB
8	F	86	T	tub1	pT3	pN1	M0	IIIB
9	M	59	S	tub2	pT3	pN1	M0	IIIB
10	F	88	S	tub1	pT3	pN1	M0	IIIB

^(1)^ Clinicopathological classification is according to UICC-TNM Classification [20]. C, cecum; A, ascending colon; T, transverse colon; S, sigmoid colon; R, rectum; pT3, tumor invading to subserosal layer; pT4a, tumor exposed to serosa; pT4b, tumor invading adjacent tissues; pN1, within three metastasized nodes; pN2, more than four metastasized nodes; M0, no distant metastasis. ^(2)^ Histopathological differentiations are according to the Japanese Classification of Colorectal Carcinoma [21]; tub1, well-differentiated adenocarcinoma; tub2, moderately differentiated adenocarcinoma; por, poorly differentiated adenocarcinoma; muc; mucinous adenocarcinoma.

**Table 3:** ELISA kits.

Target	Species	Cat#	Company
Ki67	mouse	FY-EM8749	Axel, Osaka, Japan
Ki67	human	ab253221	Abcam, Waltham, MA, USA
ALP	mouse	ab285274	Abcam, Waltham, MA, USA
ALP	human	ab285149	Abcam, Waltham, MA, USA
MUC2	mouse	M0EB0548	AssayGenie, Dublin, Ireland
MUC2	human	ab282871	Abcam, Waltham, MA, USA
αDefensin	mouse	EK11275	Signalway Antibody, Greenbelt, MA, USA
αDefensin	human	EL006657HU	Cusabio, Houston, TX, USA
Butyric acid	-	abx258338	Abbexa Ltd. Cambridge, UK

ALP, alkaline phosphatase; MUC2, mucin 2.

**Table 4:** Antibodies.

Antibody	Species	Cat#	Company
CD8	Mouse	Cat #11-0081-82	Thermo Fisher, Tokyo, Japan
CD8	Human	Cat #12-0088-42	Thermo Fisher, Tokyo, Japan
FOXP3	Mouse	#700914	Thermo Fisher, Tokyo, Japan
FOXP3	Human	#700914	Thermo Fisher, Tokyo, Japan
PDL1	Mouse	Cat #14-5982-82	Thermo Fisher, Tokyo, Japan
PDL1	Human	#14-5983-82	Thermo Fisher, Tokyo, Japan
IFNγ	Mouse	#47-7311-82	Thermo Fisher, Tokyo, Japan
IFNγ	Human	#53-7319-42	Thermo Fisher, Tokyo, Japan
CD62L	Mouse	#12-0621-82	Thermo Fisher, Tokyo, Japan
CD62L	Human	ab317478	Abcam, Waltham, MA, USA

FOXP3, forkhead box protein P3; IFNγ, interferon–gamma; PDL1, programmed cell death ligand-1.

**Table 5:** Primer sets.

Gene	Species	ID	Upper	Lower
*ACTB*	Mouse	NM_007393.5	agccatgtacgtagccatcc	ctctcagctgtggtggtgaa
*RAGE*	Mouse	L33412.1	aattgtggatcctgcctctg	aaggtaggatgggtggttcc
*TLR4*	Mouse	NM_021297.3	gcagcacctggattttcagc	ccttgacccactgcaggaat

ACTB, β-actin; RAGE, receptor for glycation end products; TLR4, Toll-like receptor-4.

## Data Availability

Data are contained within the article.

## Abbreviations

UC: ulcerative colitis; CRC, colorectal cancer; HMGB1, high-mobility group box-1; IBD, inflammatory bowel disease; CD, Crohn’s disease; TNFα, tumor necrosis factor-α; MUC2, mucin 2; αDEF, α-defensin; AOM, azoxymethane; FOXP3, forkhead box protein P3; IFN, interferon; PDL1, programmed cell death ligand-1; DSS, dextran sulfate sodium; ALP, alkaline phosphatase; BA, butyric acid; RAGE, advanced glycation end products; TLR4, Toll-like receptor 4; OXPHOS, oxidative phosphorylation.
